# Daily use of chlorine dioxide effectively treats halitosis: A meta-analysis of randomised controlled trials

**DOI:** 10.1371/journal.pone.0280377

**Published:** 2023-01-12

**Authors:** Eszter Szalai, Péter Tajti, Bence Szabó, Péter Hegyi, László Márk Czumbel, Saghar Shojazadeh, Gábor Varga, Orsolya Németh, Beata Keremi

**Affiliations:** 1 Department of Restorative Dentistry and Endodontics, Semmelweis University, Budapest, Hungary; 2 Centre for Translational Medicine, Semmelweis University, Budapest, Hungary; 3 Department of Prosthodontics, Semmelweis University, Budapest, Hungary; 4 Institute for Translational Medicine, Szentágothai Research Centre, Medical School, University of Pécs, Pécs, Hungary; 5 Institute of Pancreatic Diseases, Semmelweis University, Budapest, Hungary; 6 Department of Periodontology, Semmelweis University, Budapest, Hungary; 7 Faculty of Dentistry, Semmelweis University, Budapest, Hungary; 8 Department of Oral Biology, Semmelweis University, Budapest, Hungary; 9 Department of Community Dentistry, Semmelweis University, Budapest, Hungary; University of Catania: Universita degli Studi di Catania, ITALY

## Abstract

**Objectives:**

We aimed to conduct a systematic review on published data in order to investigate the efficacy of mouthwash products containing chlorine dioxide in halitosis.

**Study design:**

Systematic review and meta-analysis

**Methods:**

Our search was conducted on 14th October 2021. We searched the following electronic databases: MEDLINE, Embase, Scopus, Web of Science, and CENTRAL. We analysed data on adults with halitosis, included only randomised controlled trials and excluded *in vitro* and animal studies. The interventional groups used chlorine dioxide, and the comparator groups used a placebo or other mouthwash. Our primary outcomes were changes in organoleptic test scores (OLS) and Volatile Sulfur Compound (VSC) levels from baseline to the last available follow-up.

**Results:**

We found 325 articles in databases. After the selection process, ten articles were eligible for qualitative synthesis, and 7 RCTs with 234 patients were involved in the meta-analysis. Our findings showed a significant improvement in the parameters of the chlorine dioxide group compared to the placebo group in OLS one-day data (mean difference (MD): -0.82; 95% confidence intervals (95% CIs): [-1.04 –-0.6]; heterogeneity: I^2^ = 0%, p = 0.67); and one-week OLS data (MD: -0.24; 95% CIs: [-0.41 –-0.07]; I^2^ = 0%, p = 0.52); and also changes in H_2_S one-day data (standardised mean difference (SMD): -1.81; 95% CIs: [-2.52 –-1.10]); I^2^ = 73.4%, p = 0.02).

**Conclusion:**

Our data indicate that chlorine dioxide mouthwash may be a good supportive therapy in oral halitosis without known side effects.

## Introduction

Halitosis or bad breath, defined as ’’malodor with intensity beyond a socially acceptable level, perceived’’ [1], is an unpleasant condition that most people experience or notice in others. Halitosis may result in higher anxiety levels, feelings of inadequacy, depression, sensitivity, anger, and stress [2].

Oral microbial putrefaction of proteins is the leading cause of Type 1(oral) halitosis [3]. This process results in the formation of volatile sulfur compounds (VSCs). The main VSCs involved in oral halitosis are hydrogen sulfide (H_2_S), methyl mercaptan (CH_3_SH), and dimethyl sulfide ((CH_3_)_2_S) [4]. The first two compounds are responsible for approximately 90% of VSCs [5]. These VSCs are mainly produced by gram-negative anaerobic oral bacteria (*Aggregatibacter actinomycetemcomitans*, *Porphyromonas gingivalis*, *Fusobacterium nucleatum*, *Tannerella forsythia*, and *Treponema denticola*) [6–8] from sulfur-containing amino acids such as cysteine, cystine, and methionine [4, 9].

There is still no evidence-based, definitive treatment protocol for bad breath. As mentioned in a systematic review by Wylleman et al. [10], it was proved that tongue cleaning is effective in reducing oral malodor in addition to toothbrushing. If measures do not help and the supposed cause was also treated well (e.g., periodontitis), further treatment might be necessary [11, 12]; namely, the use of mouthwashes (e.g. Halita™, meridol® [11], stannous fluoride and zinc lactate [13] or chlorine dioxide mouthwashes [14]) or probiotics (e.g. *Lactobacillus salivarius*, *Lactobacillus reuteri* [15–17], *Bifidobacterium lactis* and *Lactobacillus acidophilus* [18]). There are various types of mouthwash on the market, and people spend millions of dollars annually on anti-malodor mouthwash products [19]. Chlorhexidine-containing mouthwashes are considered to be the gold standard [20] mouthwashes. Although they are effective, they have several side effects [20, 21]. There is an obvious need to find a mouthwash that treats halitosis effectively and without side effects.

Chlorine dioxide (ClO_2_) is a selective oxidizing agent [22]. Unlike other oxidants, it reacts poorly with most substances in living organisms [22]. However, it rapidly responds with three amino acids: cysteine, tyrosine, and tryptophan. The anti-halitotic activity of ClO_2_ is primarily an antibacterial effect due to its reactions with the three amino acids mentioned above and their acid residues in proteins and peptides [22]. Furthermore, it oxidises the precursors of VSCs [23, 24]. These antimicrobial mouthwashes are mainly effective against Type 1 halitosis.

The aqueous chlorine dioxide solution [25] is widely used in medicine for disinfection of intraoral areas [26–29], without any recorded side effects [30]. Several studies have already been conducted to investigate chlorine dioxide mouthwashes in halitosis [29, 31–34]; however, these individual studies lack high power, and there is imprecision in the data. Cochrane review [35] did not find sufficient evidence to support the effectiveness of any interventions for managing halitosis and had certain limitations in its data sets. It justifies the rationale for conducting this review.

We aimed to investigate the efficacy of chlorine dioxide mouthwashes in patients with halitosis. We hypothesised that mouthwashes containing chlorine dioxide are as efficient as other mouthwash products and more efficient than placebos in reducing oral malodor.

## Methods

### Protocol and registration

We conducted the meta-analysis according to the Preferred Reporting Items for Systematic Reviews and Meta-Analyses (PRISMA 2020) [36] statement (S1 Table) and the guidance of the Cochrane Handbook for Systematic Reviews of Interventions [37].

The protocol of this meta-analysis was registered in the International Prospective Register of Systematic Reviews (PROSPERO) under registration number CRD42021281195.

### Eligibility criteria

For eligibility, we applied the PICO (population, intervention, comparator, and outcome) framework as the reference standard. The included population were as follows: adults without systemic diseases who had bad breath; the intervention: chlorine dioxide-containing mouthwash; the comparator: other mouthwashes, placebo, or no-treatment groups; outcomes: changes in organoleptic test scores or volatile sulfur compound levels. The included population was above 18 years of age, and we did not apply any upper age limit. Bad breath was defined as OLS ≥ 1. We included only randomised controlled trials. We did not apply any language or time restrictions in our search.

We excluded in vitro and animal studies as well as patients with systemic disease or children as a population. We also excluded studies where the mouthwash with chlorine dioxide or the comparator mouthwash contained multiple active ingredients, such as chlorine dioxide and zinc.

### Information sources and search strategy

The literature search was conducted on 14th October 2021 and updated on 23rd September 2022. The search covered the following databases: MEDLINE, Embase, Scopus, Web of Science, and CENTRAL.

We used individualised search terms in different databases and examined every relevant reference list of included studies and relevant systematic reviews manually and automatically (Scopus).

### Study selection

EndNote 20 software was used for record management [38]. After duplicate removal, two investigators (E.S., P.T.) separately made the title and abstract selection to be followed by full-text selection. After the title, abstract, and full-text selection, the inter-rater agreement was measured between the investigators with Cohen’s kappa. In case of disagreement, a third author (B.K.) was also involved. If a full text could not be obtained, it was requested from the authors or libraries by E.S.

### Data collection process and data items

Two authors (E.S, P.T.) independently extracted the following data from the eligible articles and cross-checked them: population characteristics, interventions, comparator, measurement methods, and outcomes.

The primary outcome domains were organoleptic testing (OLT) scores and volatile sulfur compounds (VSCs) levels. We pooled these data from all available time points. Studies presented VSC data in either ppb or ng/10mL; some of them had total VSC data, and some separated data into H_2_S, CH_3_SH, and (CH_3_)_2_S. The ppb measurements were converted into ng/10 mL for comparison with a division of ten.

In cases of missing data, E.S. contacted the corresponding authors.

### Risk of bias in individual studies

For risk of bias assessment, the Cochrane Risk of Bias 2 Tool [39], individually-randomized, parallel-group trials, and crossover trials were used, including the following domains: bias arising from the randomisation process, bias arising from the period and the carryover effects, bias due to deviations from intended interventions, bias due to missing outcome data, bias in the measurement of the outcome, and bias in selecting the reported results. The domain of bias arising from the period and the carryover effects is the difference between the two applied Risk of Bias 2 Tools. This domain is included for crossover trials only. The two reviewers (E.S., P.T.) who made the assessments discussed and settled the disagreements. In cases of a lack of agreement, a third author was also involved.

### Effect measures

Mean-difference and standardised mean-difference meta-analyses were performed on the data with a predefined confidence interval of 95%. The mean-difference meta-analysis was performed in cases when all available data were measured using the same methods and instruments and were on the same scale. On the other hand, the standardised mean-difference meta-analysis was utilised in cases where the same parameter was measured, but the instruments differed. We applied the mean difference on the OLS data and the standardised mean difference on the VSC data because researchers used different devices to measure them. Studies that did not include Standard Deviations (SD) for either measurement and those with SDs that were not computable from the OLS data or with the latter mentioned methods were excluded from the meta-analyses and were used only for the ‘qualitative results’ part of this study. We also calculated the changes in the outcome data in various periods; this was the criteria to form subgroups. The OLS subgroups demonstrate one-day, one-week, and two-week data separately.

In the case of crossover studies, only the results of the first phases were utilised as a conservative and cautious approach. It was thus ensured that there was no distortion due to the inclusion of dependent study populations.

In cases where the standard deviation of changes in the measurements for the different follow-up times was not given, Cochrane guidelines [37] were used. When researchers gave only a confidence interval (CI) for the change, we divided the difference between the upper and lower CI limits by 3.92 (the value for 95% CI) [37]. When there was an available SD of the change in any study, a correlation coefficient was calculated using the SD value for the intervention and the control groups of the study, and the missing SDs of the other studies were calculated using this correlation coefficient value [37].

### Synthesis methods

The weight of each study in the meta-analysis was based on its standard deviations and sample size. Larger SDs or a smaller sample size resulted in a lower weight assigned to the specific study. In contrast, studies with small SDs or a high sample size received higher weights in the analyses. Statistical heterogeneity was calculated using the I-squared test. For the meta-analyses, random-effects models were used, as the population of the studies was expected to be heterogeneous. All statistical analyses were carried out using R-statistics [40] and its "meta" package. The results of the meta-analyses were presented using forest plots.

### Reporting bias assessment

Funnel plot analyses and heterogeneity analysis could not be appropriately performed due to the low number of articles.

### Certainty assessment

The certainty assessment was evaluated according to the GRADE Handbook [41]; we performed the summary of findings table with the GRADEpro [42] tool. Two reviewers (E.S., P.T.) assessed the certainty of evidence individually, the discrepancies were discussed, and consensual decisions were made.

## Results

### Study selection

**Fig 1:**
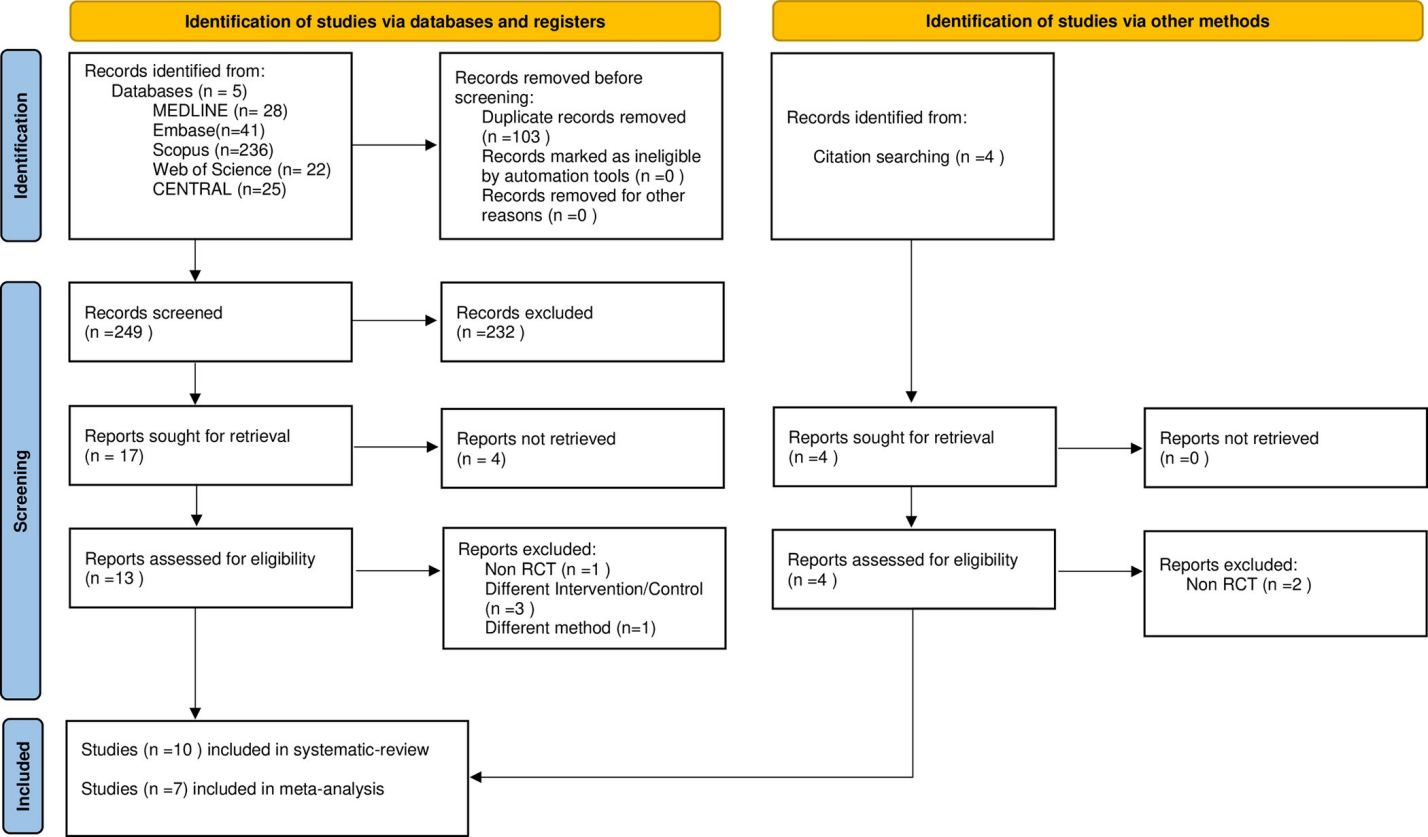
Prisma 2020 flow diagram of the screening and selection process.

352 articles were downloaded: MEDLINE (n = 28), Embase (n = 41), Scopus (n = 236), Web of Science (n = 22), and CENTRAL (n = 25). After duplicate removal, we had 249 articles (Fig 1).

For full-text selection, 17 abstracts were selected. Eight articles remained after we compared our choices from the eligible full texts. The first Cohen’s kappa was 1, and the second was 0.88. Four articles were excluded because the full texts were not available [29, 33, 34, 43]; one report was excluded [44] because it was not a randomized controlled trial; three other articles were excluded because the intervention [31, 45] or a comparator [32] contained Zn, an active component not considered in our analysis. Another study was excluded because cysteine was used to induce halitosis [46]. We found four articles [47–50] by citation search. Two were eligible for full texts [48, 50]. The other two articles from the citation search were not RCTs [47, 49] and were thus excluded.

After the selection process, a total of ten articles were included in the qualitative synthesis [14, 48, 50–57], and seven in the quantitative synthesis. Three articles could not be included in the statistical analysis because we did not have enough comparable data [14, 50, 54].

### Characteristics of the included studies

Key characteristics of the included studies are presented in Table 1. Placebo was used in the comparator groups, except for one study [50], where it was chlorhexidine.

**Table 1:** Main characteristics of the included studies.

Publication Data			Population	Age			N0 of Patients	Sex (Female/ Male)	N° of Patients/ Groups	Care product	Main content	Outcomes			Time points
First Author / Year of Publication	Country	Study Design		Mean	SD	Range						OLT	VSC	Other	
Shinada et al. 2010	Japan	RCT, double-blind, crossover	healthy	22,9	6,2	19–38	15	0/15	8	ClO_2_ Fresh	0.1% ClO_2_	yes	GC8A gas chromato-graph	PI, GI, TCI, TDI, Resting saliva, F.n., T.d., T. f., P.g.	Baseline 1-week
									7	Placebo					
Aung et al. 2015	Myanmar	RCT, single-blind, parallel	healthy VSCs more than 250 ppb	19,8	2,9	18–30	30	0/30	15	Fresh	ClO_2_	no	Breathtron	DMF, DI, BOP, TC, Ph of saliva, flow	Baseline, 1, 2, 3, 4, 5 week
									15	just tooth brushing					
Pham et al. 2018	Vietnam	RCT, double-blind, crossover	healthy students, OM>2	NA	NA	19–23	39	19/20	17	Thera-Breath®	0.1% ClO_2_	yes	Oral-Chroma	PI, GI, TCI, T.f., F.n., P.g., T.d, salivary pH and flow rate	Baseline, 12-hour, 2-week
									22	placebo	sodium chloride 0.9%				
Peruzzo et al. 2007	Brasil	RCT double-blind, crossover	dental students	NA	NA	18–25	14	08/06	7	SaudBucal®	0.1% ClO_2_	no	Halimeter	NA	Baseline, 4-day
									7	placebo	NA				
Shetty et al. 2013	India	RCT, double-blind, crossover	healthy men	NA	NA	18–35	18	0/18	9	Thera-Breath®	0.1% stabilised ClO_2_	no	Halimeter	PI, GI	Baseline, 7-day
									9	CHX	chlorhexidine 0.2%				
Grootveld et al. 2018	UK	RCT, double blind, crossover	healthy patients	NA	NA	24–55	30	13/17	NA		0.10% NaClO_2_	no	Oral-Chroma	NA	Baseline, 0,33, 4, 8 and 12-hour
									NA	H2O					
Shinada et al. 2008	Japan	RCT, double-blind, crossover	healthy men	22,9	6,2	19–38	15	0/15	8	ClO2 fresh	0.16% NaClO_2_	yes	gas chromato-graph	DMF, PI, GI	Baseline, 0,5, 2, 4-hour
									7	Placebo					
Bestari et al. 2017	Indonesia	RCT, single-blind	NA	NA	NA	NA	40	NA	20	Oxyfresh® “Oxygene® "	ClO_2_	yes	Oral-Chroma	NA	Baseline, 0,5, 2, 4, 6-hour
									20	Placebo	dest. water				
Lee et al. 2021	USA	RCT, double-blind, crossover	healthy patients, 4.5>OM>2.6	39,4	13,3	21–65	48	34/14	24	CloSYS	0.1% stabilized ClO_2_	yes	no	NA	Baseline, 1,2,3-week
									24	Placebo					
Lee et al. 2018	USA	RCT, double-blind, crossover	healthy patients, 4.5>OM>2.6	45,4	13,5–14,4	21–65	48	30/18	23	CloSYS	0.1% ClO_2_	yes	no	NA	Baseline, 0,5, 2, 4-hour
									25	Placebo					

RCT: randomised clinical trials; OLS: organoleptic test scores; SD: standard deviation; ClO_2_: chlorine dioxide; NaClO_2_: sodium chlorite; NA: not available, PI: Plaque index; GI: Gingival index, TCI: Tongue coating index; TDI: Tongue discolouration index, DMF: number of decayed, filled, and missing teeth T.f.: Tannerella forsythia, F.n.: Fusobacterium nucleatum; P.g.: Prphyromonas gingivalis, T.d.: Treponema denticola; S.m.: Streptococcus mutans

Four studies did not involve women because their menstruation cycles could influence the results [14, 50, 56, 57]. All the studies were written in English. We included two articles by Lee et al. [52, 53]; the corresponding author confirmed that the applied populations differed. After that, we summarised one-day, one-week, and two-week data. In the VSC 1-week and 2-week data, we did not have enough comparative articles, so we had to exclude three articles from the quantitative synthesis [14, 50, 54]. The one-day follow-up patients used the experimental mouthwashes in the morning of the measurement day, and on the one-week and two-week follow-ups, they used them twice a day. No other intervention was allowed for the patients.

All of the included studies used the six-point OLS scale [58]. The organoleptic method measures the intensity of halitosis from 0 to 5, where 0 means no malodor, and 5 indicates very severe malodor [58].

We did not analyse secondary outcomes because Kerémi et al. [27] further performed a meta-analysis of our secondary outcomes.

### Results of individual studies and the results of the synthesis

**Fig 2:**
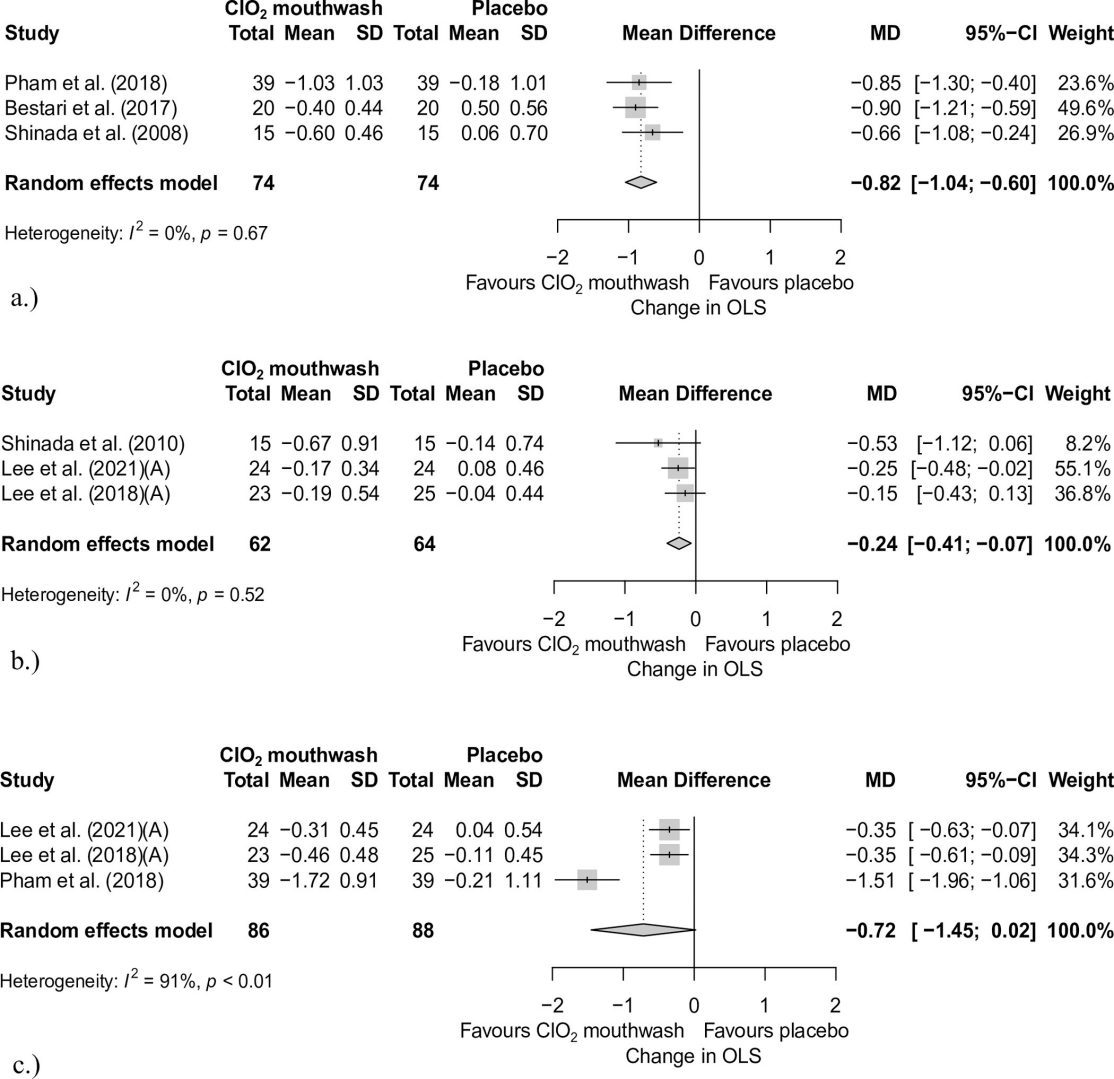
Forest plot analysis of the changes of organoleptic measurement. a. between baseline and within one day with and without ClO_2_ mouthwash. b. between baseline and within one week with and without ClO_2_ mouthwash. c. between baseline and within two weeks with and without ClO_2_ mouthwash. MD: Mean difference; CI: Confidence interval; SD: Standard deviation; ClO_2_: chlorine dioxide; OLS: organoleptic test scores.

Altogether, 234 patients were included in the quantitative analysis. None of the studies reported any side effects experienced by the patients. Our forest plots show a significant improvement in the parameters of the chlorine dioxide group compared to the control (placebo) group in organoleptic scores (Fig 2A and 2B).

One-day OLS data were pooled from three articles [51, 55, 56] after 4, 6, and 12 hours. The results show the effectiveness of chlorine dioxide in the one-day data (mean difference (MD): -0.82; 95% confidence intervals (95% CIs): [-1.04 –-0.6]; heterogeneity: I^2^ = 0%, p = 0.67) (Fig 2A).

One-week OLS data pooled from three articles [52, 53, 57] and the results are also in favor of the experimental group (MD: -0.24; 95% CI: [-0.41 –-0.07]); I^2^ = 0%, p = 0.52) (Fig 2B).

Two-week OLS data were collected from three articles [52, 53, 55], and the results also show the effect of chlorine dioxide-containing mouthwashes in halitosis (MD: -0.72; 95% CI: [-1.45–0.02]; I^2^ = 91%, p< 0.01) (Fig 2C).

**Fig 3:**
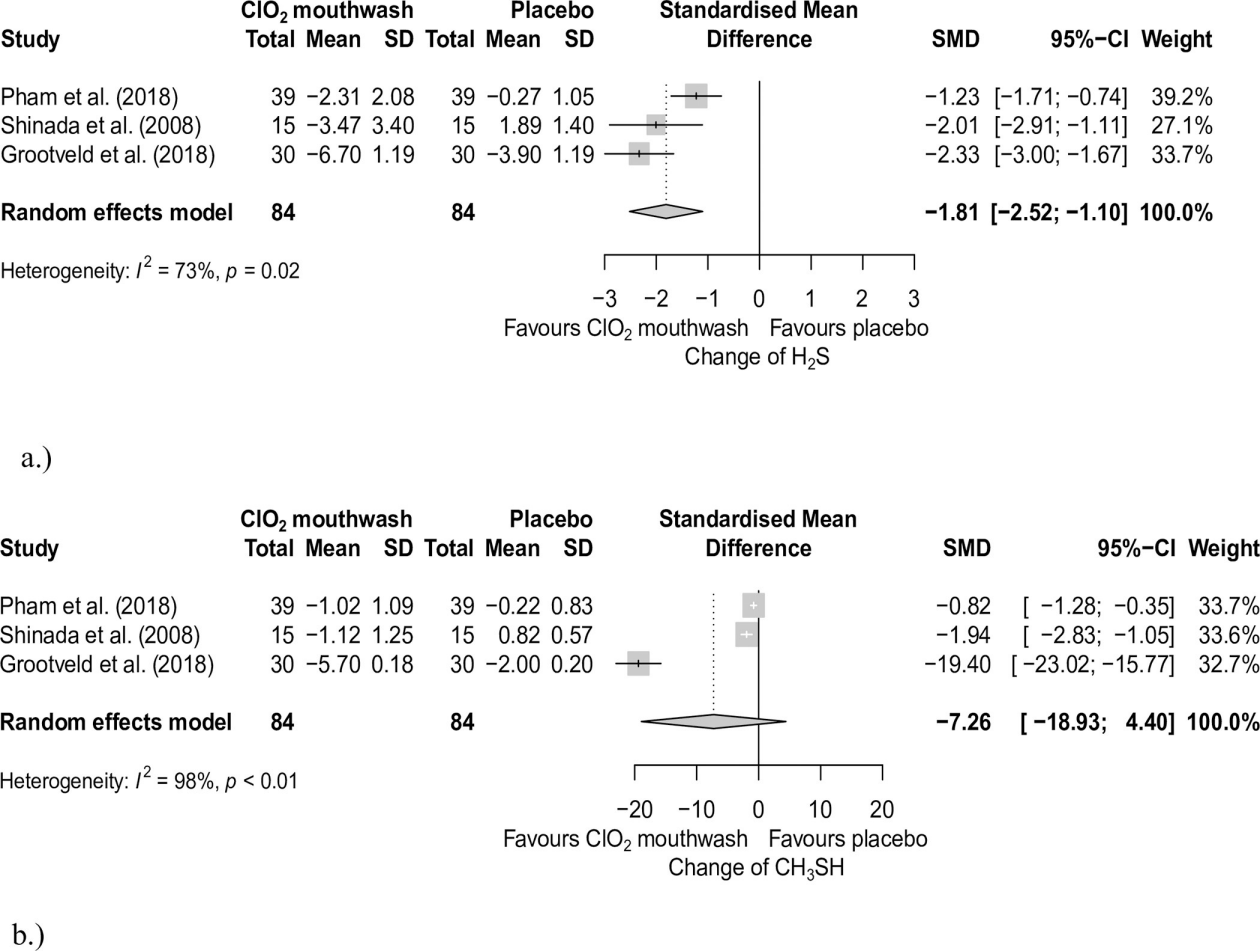
a. Forest plot analysis of the changes of hydrogen sulfide concentration between baseline and within one day with and without ClO_2_ mouthwash. b. Forest plot analysis of the changes of methyl mercaptan within concentration between baseline and one day with and without ClO_2_ mouthwash. SMD: Standardized mean difference; CI: Confidence interval; SD: Standard deviation; ClO_2_: chlorine dioxide.

Changes in H_2_S and CH_3_SH one-day data were pooled from three articles [48, 55, 57]. We also found significant differences in H_2_S data (standardized mean difference: (SMD): -1.81; 95% CI: [-2.52 –-1.10]; I^2^ = 73.4%, p = 0.02) (Fig 3A), but we did not find significant differences in the CH_3_SH one-day data (SMD: -7.26; 95% CI: [-18.93–4.4]; I^2^ = 98.0%, p< 0.01) (Fig 3B).

### Risk of bias in studies

All the included crossover studies [48, 50, 52–57] had a low risk of bias in Domains 1–4, except for two [48, 50]. We evaluate bias arising from the period, the carryover effects, and deviations from the intended interventions, such as some concerns because we do not have any information about it in the article by Grootveld et al. [48]. Shetty et al. [50] did not give detailed information about randomisation, so we evaluated some concerns in the first domain. We considered two articles as cluster-randomized trials [14, 51]. Bestari et al. [51] received some concerns about the risk of bias because of missing information in the related question due to deviations from intended interventions and bias in selecting the reported result. In Domain 5, the bias of selection of the reported results, we evaluated all the studies as of some concern because even if they published the trial protocols, these did not contain the pre-specified analysis plan. All included studies represented good quality, but we had to evaluate the overall risk as of some concern because of Domain 5 (S1 and S2 Figs).

### Publication bias and heterogeneity

The heterogeneity might not be important in the one-day and one-week data; however, the two-week OLS data may represent considerable heterogeneity. There was substantial statistical heterogeneity in H_2_S data and considerable statistical heterogeneity in CH_3_SH data.

### Certainty of evidence

The certainty assessment of the investigated outcomes displays very low to moderate certainty of evidence (S1 Table). We had to downgrade our results, primarily because of statistical heterogeneity, the risk of bias assessment, and imprecision. The statistical estimation caused a higher confidence interval, which increased imprecision.

## Discussion

The study aimed to investigate the efficacy of mouthwashes containing chlorine dioxide. On the basis of our results, mouthwashes containing chlorine dioxide effectively reduce the level of halitosis against placebos in OLS and VSCs measurements in the short term in one-day and one-week data. In one-day and two-week data, chlorine dioxide decreased the halitosis level by almost one in a five-degree scale, which means that bad breath decreased. Significant results in hydrogen sulfide data support it because it is the main component of oral malodor. Articles that could not include quantitative synthesis had a similar conclusion [14, 32, 54]. The one eligible article with a mouthwash comparator containing chlorhexidine showed chlorine dioxide to be almost as efficient as chlorhexidine [50], but it was unfeasible to carry out a meta-analysis. A few patients reported an unpleasant mouthwash taste [50], but researchers concluded that this problem was treatable with a masking agent [50]. Moreover, patients did not experience side effects in the short term (2 weeks) and when using lower concentrations (0.1% chlorine dioxide). Similar results were found about the adverse effect of chlorine dioxide in another systematic review [59]. However, the experimental mouthwash’s overuse (with 24–48 h incubation time) can be cytotoxic or apoptotic on human cells [60].

Heterogeneity in the studies included could originate from various factors. The study designs and protocols were slightly different, and we included studies with various follow-up periods. Furthermore, in the case of moderate or substantial heterogeneity, we supposed other confounding factors besides the low number of studies. We hypothesised that the reason for a moderate statistical heterogeneity was the difference in rinsing protocols. Pham et al. [55] instructed their patients to rinse with 15 mL of mouthwash for 30 sec, then spit and continue to gargle with 15 mL of mouthwash for 15 sec, whereas Lee et al. [52, 53] instructed patients to gargle with 15 mL for 30 sec only. The reason for the extremely high statistical heterogeneity of CH_3_SH data may be the longer compulsory mouth closing before measurement, which Grootveld et al. [48] applied for 5 minutes, while in the other studies it was applied for 3 minutes only. Further explanations may be that methyl mercaptan concentration depends mainly on *Porphyromonas gingivalis* [61], and that racial differences can cause differences in bacterial composition [62]; two articles [55, 56] are from Asia, and one is from the UK [48]. Furthermore, elevated CH_3_SH concentration in periodontal disease [63, 64] is well-known, but Grootveld et al. [48] exclude periodontopathic patients. However, due to the low number of included studies, our assumptions to explain the heterogeneity of measurement readings should be handled with care.

Our investigations proved that out of VSCs, chlorine dioxide reduces mainly hydrogen sulfide. Furthermore, H_2_S may predict further progress and severe conditions [65], like periodontitis, and oxidative stress [65, 66]. Takeshita et al. [67] suggest that higher CH_3_SH or H_2_S levels originate from different bacteria, and it is unnecessary to separate VSCs in order to check the overall effect. We believe that targeted therapy facilitates patient well-being. Additionally, Ademovski et al. [32] mentioned that chlorine dioxide primarily reduces dimethyl sulfide. We did not have a synthesised result from dimethyl sulfide data. However, we are certain that dimethyl sulfide is not the main component of VSCs.

As we know, we do not have an evidence-based treatment protocol for malodor. We agree with the systematic review by Nagraj et al. [35], who found low-certainty evidence to support the effectiveness of interventions for managing halitosis compared to a placebo or control for the OLT [35, 68]. On the basis of our results, mouthwash containing chlorine dioxide may be effective in halitosis and is free of known side effects. The efficacy is visible both on OLT and VSC data when compared to another meta-analysis conducted on probiotics, which reduced only OLT results [69]. The side effects of chlorhexidine and alcohol-containing mouthwashes are well-known [20, 70]. Moreover, another meta-analysis [71], which investigated the carcinogenic effect of alcohol-containing mouthwashes, did not find sufficient evidence. However, it concluded that patients should minimise their long-term use. The selective toxicity of chlorine dioxide, based on its mechanism of action [22], is the most significant point supporting its clinical benefits over other disinfectants [72]. The cost of this therapy is similar to or a bit higher than therapy with other mouthwashes, although it depends mainly on the brand of the selected mouthwash. We think our results are promising, and our findings suggest that chlorine dioxide is a valid alternative. For the above reasons, we suggest using mouthwashes containing chlorine dioxide rather than chlorhexidine against intraoral halitosis.

### Strengths and limitations

Our meta-analysis’s strengths are the pre-registered, well-documented methodology and the fact that all the included studies are RCTs. Another strength is that we had organoleptic measurement data in more time points, which can follow the mid-term effects. We also think that separated VSC results help to understand chlorine dioxide’s efficacy better.

The main limitation of our paper is the relatively small number of included studies. Other significant limitation, there are not enough comparable results with other mouthwashes containing active ingredients. Four studies could not be retrieved. We could not perform a meta-analysis from the total VSC data, and long-term effect follow-ups are missing. Furthermore, physiological and pathological halitosis could not be adequately differentiated when conducting the meta-analysis. Due to the low number of studies, the results of the analyses must be handled with caution, as the inclusion of further studies could easily change the results acquired.

### Implications for research

We believe our findings will facilitate further investigations of other mouthwashes in halitosis therapy. We suggest that further studies should present their data also in total VSCs with SD so as to be make them comparable because if we summarise the H_2_S, CH_3_SH, and (CH_3_)_2_S data, we lose the SD. Furthermore, it is necessary to define the minimally important difference data (MID) to conclude whether the statistical evidence is in line with the clinical evidence as well.

### Implications for practice

Patients with oral halitosis are easily treatable with side-effect-free chlorine dioxide mouthwashes. Based on our investigation, chlorine dioxide-containing mouthwashes may be preferable to other mouthwashes, and consequently, they can be the first choice of dentists and patients. We also think chlorine dioxide could play a prominent role in targeted therapy for H_2_S.

## Conclusion

The findings suggest that chlorine dioxide mouthwashes should receive a more prominent role in the supportive therapy of oral halitosis. Our results show that it is effective against halitosis in the short term compared to the placebo. Especially patients with an elevated H_2_S level can benefit from a targeted treatment because chlorine dioxide demonstrates greater efficacy in that compound.

## Supporting information

S1 Fig Risk of bias-2 assessment of the included crossover studies. The domains and the overall risk of bias were marked using the following traffic light system: red signified high risk, yellow indicated some concerns, and green represented a low risk of bias.
(PDF)

S2 Fig Risk of bias-2 assessment of the included parallel study. The domains and the overall risk of bias were marked using the following traffic light system: red signified high risk, yellow indicated some concerns, and green represented a low risk of bias.
(PDF)

S1 Table PRISMA checklist. From: Page MJ, McKenzie JE, Bossuyt PM, Boutron I, Hoffmann TC, Mulrow CD, et al. The PRISMA 2020 statement: an updated guideline for reporting systematic reviews. BMJ 2021;372:n71. DOI: 10.1136/bmj.n71s. For more information, visit: http://www.prisma-statement.org/.
(DOCX)

S2 Table Summary of evidence table. **CI:** confidence interval; **MD:** mean difference; **SMD:** standardised mean difference. Explanations. a. Statistical heterogeneity I^2^ = 73%. b. Statistical heterogeneity I^2^ = 91%. c. Statistical heterogeneity I^2^ = 96%. d. Funnel plot analysis was performed.
(DOCX)

S3 Table Data included in the meta-analysis. Note: CI: Confidence interval; SD: Standard deviation.
(PDF)
