# Supplementary material for: Daily use of chlorine dioxide effectively treats halitosis: A meta-analysis of randomised controlled trials
Source: PLoS One. 2023 Jan 12;18(1):e0280377. doi: 10.1371/journal.pone.0280377 (PMC9836286; doi:10.1371/journal.pone.0280377)
Supplement: S1 Fig — The domains and the overall risk of bias were marked using the following traffic light system: red signified high risk, yellow indicated some concerns, and green represented a low risk of bias. (PDF) [file pone.0280377.s001.pdf]

Intention-to-treat

Unique ID	Study ID	Experimental	Comparator	Outcome	Weight	D1	DS	D2	D3	D4	D5	Overall
Clo2_c38	Grootveld et al. 20	ClO2	Placebo	VSC ((CH3)2S)	1	+	!	!	+	+	!	!
Clo2_c37	Grootveld et al. 20	ClO2	Placebo	VSC (CH3SH)	1	+	!	!	+	+	!	!
Clo2_c13	Grootveld et al. 20	ClO2	Placebo	VSC (H2S)	1	+	!	!	+	+	!	!
Clo2_c11	Lee at al. 2018	ClO2	Placebo	OLT	1	+	+	+	+	+	!	!
Clo2_c31	Lee at al. 2018	ClO2	Placebo	OLT	1	+	+	+	+	+	!	!
Clo2_c32	Lee at al. 2018	ClO2	Placebo	OLT	1	+	+	+	+	+	!	!
Clo2_c33	Lee at al. 2018	ClO2	Placebo	OLT	1	+	+	+	+	+	!	!
Clo2_c12	Lee et al 2021	ClO2	Placebo	OLT	1	+	+	+	+	+	!	!
Clo2_c34	Lee et al 2021	ClO2	Placebo	OLT	1	+	+	+	+	+	!	!
Clo2_c35	Lee et al 2021	ClO2	Placebo	OLT	1	+	+	+	+	+	!	!
Clo2_c36	Lee et al 2021	ClO2	Placebo	OLT	1	+	+	+	+	+	!	!
Clo2_c3	Peruzzo et al. 2006	ClO2	placebo	VSC	1	+	+	+	+	+	!	!
Clo2_c14	Peruzzo et al. 2006	ClO2	placebo	VSC	1	+	+	+	+	+	!	!
Clo2_c9	Pham et al. 2018	ClO2	Placebo	OLT	1	+	+	+	+	+	!	!
Clo2_c24	Pham et al. 2018	ClO2	Placebo	OLT	1	+	+	+	+	+	!	!
Clo2_c25	Pham et al. 2018	ClO2	Placebo	OLT	1	+	+	+	+	+	!	!
Clo2_c27	Pham et al. 2018	ClO2	placebo	VSC (CH3SH)	1	+	+	+	+	+	!	!
Clo2_c28	Pham et al. 2018	ClO2	placebo	VSC (CH3SH)	1	+	+	+	+	+	!	!
Clo2_c30	Pham et al. 2018	ClO2	placebo	VSC (CH3SH)	1	+	+	+	+	+	!	!
Clo2_c10	Pham et al. 2018	ClO2	placebo	VSC (H2S)	1	+	+	+	+	+	!	!
Clo2_c26	Pham et al. 2018	ClO2	placebo	VSC (H2S)	1	+	+	+	+	+	!	!
Clo2_c29	Pham et al. 2018	ClO2	placebo	VSC (H2S)	1	+	+	+	+	+	!	!
Clo2_c8	Shetty et al. 2013	ClO2	CHX	VSC	1	!	+	+	+	+	!	!
Clo2_c23	Shetty et al. 2013	ClO2	CHX	VSC	1	!	+	+	+	+	!	!
Clo2_c4	Shinada et al. 2010	ClO2	Placebo	OLT	1	+	+	+	+	+	!	!
Clo2_c15	Shinada et al. 2010	ClO2	Placebo	OLT	1	+	+	+	+	+	!	!
Clo2_c6	Shinada et al. 2008	ClO2	Placebo	OLT	1	+	+	+	+	+	!	!
Clo2_c17	Shinada et al. 2008	ClO2	Placebo	OLT	1	+	+	+	+	+	!	!
Clo2_c19	Shinada et al. 2008	ClO2	Placebo	VSC ((CH3)2S)	1	+	+	+	+	+	!	!
Clo2_c22	Shinada et al. 2008	ClO2	Placebo	VSC ((CH3)2S)	1	+	+	+	+	+	!	!
Clo2_c18	Shinada et al. 2008	ClO2	Placebo	VSC (CH3SH)	1	+	+	+	+	+	!	!
Clo2_c21	Shinada et al. 2008	ClO2	Placebo	VSC (CH3SH)	1	+	+	+	+	+	!	!
Clo2_c7	Shinada et al. 2008	ClO2	Placebo	VSC (H2S)	1	+	+	+	+	+	!	!
Clo2_c20	Shinada et al. 2008	ClO2	Placebo	VSC (H2S)	1	+	+	+	+	+	!	!
Clo2_c5	Shinada et al. 2010	ClO2	placebo	VSC	1	+	+	+	+	+	!	!
Clo2_c16	Shinada et al. 2010	ClO2	placebo	VSC	1	+	+	+	+	+	!	!

+	Low risk
!	Some concerns
-	High risk

D1	Randomisation process
DS	Bias arising from period and carryover effects
D2	Deviations from the intended interventions
D3	Missing outcome data
D4	Measurement of the outcome
D5	Selection of the reported result
