# Supplementary material for: Daily use of chlorine dioxide effectively treats halitosis: A meta-analysis of randomised controlled trials
Source: PLoS One. 2023 Jan 12;18(1):e0280377. doi: 10.1371/journal.pone.0280377 (PMC9836286; doi:10.1371/journal.pone.0280377)
Supplement: S2 Fig — The domains and the overall risk of bias were marked using the following traffic light system: red signified high risk, yellow indicated some concerns, and green represented a low risk of bias. (PDF) [file pone.0280377.s002.pdf]

Intention-to-treat

Unique ID	Study ID	Experimental	Comparator	Outcome	Weight	D1	D2	D3	D4	D5	Overall
clo2_1	Bestari et al 2016	ClO2	Placebo	VSC	1	+	!	+	+	!	!
clo2_2	Aung et al. 2014	ClO2	Tongue cleaning	VSC	1	+	+	+	+	!	!
clo2_3	Bestari et al 2017	ClO2	Placebo	VSC	1	+	!	+	+	!	!
clo2_4	Bestari et al 2017	ClO2	Placebo	OLT	1	+	!	+	+	!	!
clo2_5	Bestari et al 2017	ClO2	Placebo	OLT	1	+	!	+	+	!	!

+ Low risk

! Some concerns

- High risk

D1 Randomisation process

D2 Deviations from the intended interventions

D3 Missing outcome data

D4 Measurement of the outcome

D5 Selection of the reported result
