# Supplementary material for: Daily use of chlorine dioxide effectively treats halitosis: A meta-analysis of randomised controlled trials
Source: PLoS One. 2023 Jan 12;18(1):e0280377. doi: 10.1371/journal.pone.0280377 (PMC9836286; doi:10.1371/journal.pone.0280377)
Supplement: S2 Table — CI: confidence interval; MD: mean difference; SMD: standardised mean difference. Explanations. a. Statistical heterogeneity I2 = 73%. b. Statistical heterogeneity I2 = 91%. c. Statistical heterogeneity I2 = 96%. d. Funnel plot analysis was performed. (DOCX) [file pone.0280377.s004.docx]

| **Certainty assessment** | | | | | | | **№ of patients** | | **Effect** | | **Certainty** | **Importance** |
| --- | --- | --- | --- | --- | --- | --- | --- | --- | --- | --- | --- | --- |
| **№ of studies** | **Study design** | **Risk of bias** | **Inconsistency** | **Indirectness** | **Imprecision** | **Other considerations** | **Chlorine dioxide-containing mouthwashes** | **other mouthwashes or placebo** | **Relative (95% CI)** | **Absolute (95% CI)** |  |  |
| **Organoleptic test score (follow-up: mean 1 day; assessed with: dentist's perception; Scale from 0 to 5)** | | | | | | | | | | | | |
| 3 | randomized trials | serious^a^ | not serious | not serious | serious^b^ | none | 74 | 74 | - | MD **0.82 lower** (1.04 lower to 0.6 lower) | ⨁⨁◯◯ Low | CRITICAL |
| **Volatile sulfur compounds: Hydrogen sulfide (follow-up: mean 1 day; assessed with portable VSCs monitor or portable gas chromatograph)** | | | | | | | | | | | | |
| 3 | randomized trials | serious^a^ | serious^c^ | not serious | very serious^b^ | none | 84 | 84 | - | SMD **1.81 SD lower** (2.52 lower to 1.1 lower) | ⨁◯◯◯ Very low | CRITICAL |
| **Organoleptic test score (follow-up: mean 1 week; assessed with: dentist's perception; Scale from 0 to 5)** | | | | | | | | | | | | |
| 3 | randomized trials | not serious | not serious | not serious | serious^b^ | none | 62 | 64 | - | MD **0.24 lower** (0.41 lower to 0.07 lower) | ⨁⨁⨁◯ Moderate | CRITICAL |
| **Organoleptic test score (follow-up: mean 2 weeks; assessed with: dentist's perception; Scale from 0 to 5)** | | | | | | | | | | | | |
| 3 | randomized trials | not serious | very serious^d^ | not serious | very serious^b^ | none | 86 | 88 | - | MD **0.72 lower** (1.45 lower to 0.02 higher) | ⨁◯◯◯ Very low | CRITICAL |
| **Volatile sulfur compounds: Methyl mercaptan (follow-up: mean 1 day assessed with: portable VSCs monitor, or portable gas chromatograph)** | | | | | | | | | | | | |
| 3 | randomized trials | serious | very serious^e^ | not serious | serious^b^ | none | 84 | 84 | - | SMD **7.26 SD lower** (18.93 lower to 4.4 higher) | ⨁◯◯◯ Very low | CRITICAL |

**Table S2: Summary of evidence table**

**CI:** confidence interval; **MD:** mean difference; **SMD:** standardized mean difference

#### Explanations

a. Two domains received some concerns.

b. The estimated confidence intervals were high

c. Statistical heterogeneity I^2^=73%

d. Statistical heterogeneity I^2^=91%

e. Statistical heterogeneity I^2^=96%
