# Supplementary material for: Daily use of chlorine dioxide effectively treats halitosis: A meta-analysis of randomised controlled trials
Source: PLoS One. 2023 Jan 12;18(1):e0280377. doi: 10.1371/journal.pone.0280377 (PMC9836286; doi:10.1371/journal.pone.0280377)
Supplement: S3 Table — Note: CI: Confidence interval; SD: Standard deviation. (PDF) [file pone.0280377.s005.pdf]

ORGANOLEPTIC	baseline						1 week					
	intervention			control			intervention			control		
	n	mean	sd	n	mean	sd	n	mean	sd	n	mean	sd
Shinada et al. 2010	15	2,1	0,51	15	1,87	0,61	15	1,43	0,46	15	1,73	0,56
S. S. Lee et al. 2021	24	3,08	0,38	24	3,03	0,19	24	2,92	0,31	24	3,11	0,46
S. S. Lee et al. 2021, P. II.	24	3,22	0,44	24	3,15	0,42	24	3,04	0,49	24	3,17	0,46
S. S. Lee et al. 2018	23	3,09	0,34	25	3,23	0,41	23	2,9	0,52	25	3,19	0,5
S. S. Lee et al. 2018,P. II.	24	3,14	0,46	23	3,44	0,5	24	3	0,41	23	3,18	0,61

ORGANOLEPTIC	base line						2 week					
	intervention			control			intervention			control		
	n	mean	sd	n	mean	sd	n	mean	sd	n	mean	sd
S. S. Lee et al. 2021	24	3,08	0,38	24	3,03	0,19	24	2,78	0,4	24	3,07	0,53
S. S. Lee et al. 2021, P. II.	24	3,22	0,44	24	3,15	0,42	24	2,94	0,48	24	3,13	0,4
S. S. Lee et al. 2018	23	3,09	0,34	25	3,23	0,41	23	2,64	0,48	25	3,12	0,43
S. S. Lee et al. 2018, P. II.	24	3,14	0,46	23	3,44	0,5	24	3,1	0,5	23	3,01	0,59
Pham et al. 2018	39	2,67	1	39	2,82	0,72	39	0,95	0,86	39	2,61	1,01

ORGANOLEPTIC	changes between baseline and 1 week							
		intervention			control			
	n	mean	sd	CI	n	mean	sd	CI
Shinada et al. 2010	15	-0,67			15	-0,14		
S. S. Lee et al. 2021	24	-0,17	0,34	-0,31 to -0,02	24	0,08	0,46	0,11to 0,28
S. S. Lee et al. 2021, P. II.	24	0,01	0,51	-0,2 to 0,23	24	0,08	0,47	-0,12 to 0,28
S. S. Lee et al. 2018	23	-0,19	0,54		25	-0,04	0,44	
S. S. Lee et al. 2018,P. II.	24	-0,24	0,68		23	-0,14	0,56	

ORGANOLEPTIC	changes between baseline and 2 week							
		intervention			control			
	n	mean	sd	CI	n	mean	sd	CI
S. S. Lee et al. 2021	24	-0,31	0,45	-0,5 to - 0,12	24	0,04	0,54	0,19 to 0,27
S. S. Lee et al. 2021, P. II.	24	-0,08	0,45	-0,3 to 0,1	24	0,04	0,44	-0,1 to0,20
S. S. Lee et al. 2018	23	-0,46	0,48		25	-0,11	0,45	
S. S. Lee et al. 2018, P. II.	24	-0,4	0,61		23	-0,04	0,63	
Pham et al. 2018	39	-1,72			39	-0,21		

H2S	baseline						within 1 day								
ng/10 ml	intervention			control			intervention				control				
	n	mean	sd	n	mean	sd	n	mean	sd	95%CI	n	mean	sd	95%CI	
Pham et al. 2018	39	6	5,9	39	5,98	5,34	39	3,69	4,78		39	5,71	5,21		
Bestari et al. 2017	20	0,383		20	0,342		20	0,243			20	0,655			
Shinada et al. 2008	15	5,31	4,89	15	4,88	6,61	15	1,84	1,62		15	6,77	5,96		
Grootveld et al. 2018	30	94,6		30	70,1		30	27,6		14,3	30	31,1		15,4	

CH3SH	baseline						within 1 day								
ng/10 ml	intervention			control			intervention				control				
	n	mean	sd	n	mean	sd	n	mean	sd	95%CI	n	mean	sd	95%CI	
Pham et al. 2018	39	2,55	2,07	39	2,65	2,19	39	1,53	1,92		39	2,43	1,91		
Bestari et al. 2017	20	0,426		20	0,322		20	0,297			20	0,551			
Shinada et al. 2008	15	1,42	1,48	15	1,21	1,45	15	0,3	0,28		15	2,03	1,51		
Grootveld et al. 2018	30	59,5		30	24,6		30	2,5		1,8	30	4,6		6,1	

H2S	changes between baseline and within 1 day							
ng/10 ml		intervention				control		
	n	mean	sd	CI	n	mean	sd	95%CI
Pham et al. 2018	39	-2,31	2,08		39	-0,27	1,05	
Bestari et al. 2017	20	-0,14			20	0,31		
Shinada et al. 2008	15	-3,47			15	1,89		
Grootveld et al. 2018	30	-67		6	30	-39		6

CH3SH	changes between baseline and within 1 day							
ng/10 ml		intervention				control		
	n	mean	sd	CI	n	mean	sd	95%CI
Pham et al. 2018	39	-1,02	1,09		39	-0,22	0,83	
Bestari et al. 2017	20	-0,13			20	0,23		
Shinada et al. 2008	15	-1,12			15	0,82		
Grootveld et al. 2018	30	-57		0,9	30	-20		1
